# The Prose and Poetry of a Physician’s Life

**Published:** 1893-11

**Authors:** Charles S. Webb

**Affiliations:** Atlanta, Ga.; Professor of the Practice of Medicine


ATLANTA
MEDICAL AND SURGICAL JOURNAL.

VOL. X. NOVEMBER, 1893. No. 9.

EDITED BY
LUTHER B. GRANDY, M. D., AND MILLER B. HUTCHINS, M. D.,
WITH THE CO-OPERATION OF
H. V. M. MILLER, M. D., LL. D., VIRGIL O. HARDON, M. D.,
FLOYD W. McRAE, M. D., AND ARTHUR G. HOBBS, M. D.

### *ORIGINAL COMMUNICATIONS.*

### THE PROSE AND POETRY OF A PHYSICIAN'S LIFE.

OPENING ADDRESS AT SOUTHERN MEDICAL COLLEGE, OCTOBER 3, 1893.

BY CHARLES S. WEBB, PH. B., M. D.,
Professor of the Practice of Medicine.

There is such a thing as poetic prose—where the sentiments of the human heart, the joys and sorrows, the hopes and fears are so intimately blended that you can scarcely tell where the one begins or the other ends. In the great drama of life, professional men, of all others, are set apart to see both sides of life, and of all professional men the physician *sees* more, *hears* more and *feels* more of the ups and downs, the joys and sorrows, the smiling and the weeping of the human race. He must be a flexible man, so far as human sympathy is concerned; he must be ready "to weep with those who weep and to rejoice with those who rejoice."

Alexander Pope was not a physician. He was, as one of his critics said, a poor little, deformed, drawn-up thing like an interrogation point, that could ask questions. But he was both poet and philosopher, and "Pope's Essay on Man" will be read as long as the English language is read, and that will be until the end of time.

You remember those celebrated lines:

> "Man know thyself—presume not God to scan,
> The proper study of mankind is man."

In these times, and especially within the last few years, it has been the fashion for some of the most learned in biblical study to question the inspiration of the Bible; but as one of our distinguished Americans said, "these fellows need the M. D., more than the D. D." A friend of mine, a physician, was talking with a lawyer as to the relative merits of the preacher and the doctor, and the lawyer said: "As the soul is of more value than the body, it follows that the preacher is of more benefit, because the doctor saves only the body." "Well, my friend," said the doctor (addressing the lawyer), "the best way to save your soul is to keep it in your body as long as possible, and that is what your physician is trying to do."

"The proper study of mankind is man." Yes, that is it. Man was created in the image of God. "In the image of God created He him."

It is said that Galen (or Claudius Galenus) one of the most distinguished of the ancient physicians, was converted to a belief in the existence of a great and all-wise God from the discovery and examination of a human skeleton. After studying the beautiful harmony of its mechanism, he is said to have declared that such a work was too wonderful for any but *God.* And well he might feel and think that way! He began to study *man,* and in the skeleton he saw design and wisdom infinite and power beyond all estimate set forth. What nice adjustment of the various parts! What architect with all his diagrams and books could ever invent machine like this to run for half a century or more with nothing lost from friction or decay? Here is the place, this arching cavity, in which the heart, secure in its retreat, throbbed with the pulse of life. 'Tis silent now. Did it beat more with sorrow or with joy? Did grand emotions swell within his breast, or did he plod his careless way along with stoical indifference? Alas! I cannot tell—his history is hid!

Within these hollow orbits once were placed the eyes—the "soul's interpreters." Perhaps they beamed with intellectual light—the flash from out their hidden depths revealing rare attainments in the realm of thought. Or yet it may be that, with drowsy vacancy, they showed a spirit dull and passionless. But however this may be, we know that in his ardent youth those eyes "looked love to eyes that spoke again," but now their light is fled, their ardor gone.

Here are the hands! Poor hands! What good they did I know not; perhaps they may sometimes have served unholy purposes—whose hands have not? And now this empty skull reminds me of the brain it once inclosed. What thoughts do crowd me now! The human brain, fit theme for song! 'Twas this enabled Morse to see the means by which we now compel the electric flash to bear *our* messages to distant lands. 'Twas with this light that Galileo saw the revolution of the earth upon its axis; although for this he was imprisoned and subjected to the taunts of those who walked in darkness, yet *to-day his* name is honored and oblivion has covered the names of those who sought to deprive him of his liberty.

We are speaking now of the student's first impressions of the wonderful mechanism of the human body and the wonderful achievements of *man*, who is to be his chief study. Do you remember the immortal Edward Jenner, who was scorned and scoffed at as a fanatic and a fool, when even the streets of London were filled with caricatures of "Jenner riding a cow," and now every adult and every child wears the "Jenner Coat of Arms."

And when we begin the study of the human body can we forget these things? Will they not be an inspiration to us?

The celebrated Corliss engine, which was set in motion by President Grant at the great Centennial of American Independence, was an object of interest and wonder to every person present, and also to every person who read of it in the daily press.

And yet every one who saw it, or read of it, had in his own bosom a greater mystery—*the heart,* throbbing with the pulse of life; the *brain* that enabled him *to think* about it; the *eye* with which he looked at it; the *lungs* by which he drew in the breath of life; all mysteries, greater than any ingenuity of man could possibly imagine.

The student of medicine, at the very beginning, comes into close relationship with the most interesting and the grandest part of the study of *nature*—the study of man himself, and the study of life and death.

Life and death are linked in a mystical union. We do not know what *life* is—imagination shrinks from the task, and swift-winged fancy soon returns with fruitless efforts to behold the wonders of the infinite. No poet ever can sing nor artist paint the mystery of life. No scientist can tell its seat in the human temple.

> " Between two worlds life hovers
> like a star,
> 'Twixt night and morn upon
> the horizon's verge.
> How little do we know that
> which we are,
> How less what we may be!
> The eternal surge
> Of time and tide rolls on
> and bears afar
> Our bubbles! As the old burst,
> new emerge,
> Lashed from the foam of ages,
> while the graves
> Of empires heave but like
> some passing waves."
> —*Byron.*

The study of life and death, of how to preserve the one and of how to escape the other, is a noble study, and this is *our work.* Even from the very beginning of a medical student's life, it should begin to develop his character for truth, justice, nobility and unselfishness. Let him start with this idea. The mountain stream that dances playfully along, turning the miniature mills of the children who sport upon its banks, is soon merged into the mighty river, bearing the messengers of commerce on its bosom, and deciding the situation of the great cities of the world. So it is with character. The men who have attracted the admiration of the world were moved along by easy steps of which they were scarcely aware.

In all scientific study, and especially in the study of medicine, there is abundant opportunity for developing the faculty of *thinking*, and thought, when properly directed, is "the making of a man." When Alexander H. Stephens started out for himself as a young lawyer at Crawfordville, Ga., he had a lonely time. He wrote in his diary that he spent many days in his office alone waiting for the clients that did not come, but he spent the time in hard study and in patient thinking. He was making ammunition then for the great battles of life in which he was to engage, but of which he knew nothing at that time. In a few years the opportunity was presented to him, and he was fully equipped and ready for the contest.

You will see the time, gentlemen, as perhaps all of us have, when you will be sick at heart waiting for the patients that do not come, and whilst you are very glad that the people are so healthy and none sick, yet you cannot help wishing that if there *should* be any sick they might send for you for relief. If this condition of things should ever happen to you, I beg you to have patience. Keep cool and wait. You will see many wise travelers going cheerily along through fair weather or foul, "carrying their sunshine with them." You will see many with "excelsior" on their banners, and you may catch the spirit that animates them. The silent pleadings of the representatives of true nobility will woo you to come and march with this column. Be ready, be fully equipped, and one patient may bring you a hundred more.

It is fair to compare the professions sometimes. Do you remember the celebrated S. S. Prentiss? He went to Vicksburg, thoroughly educated, but without money. He taught school and studied law, but after his first speech at the bar, he was invited by one of the leading lawyers of that city to be his partner in business. From that hour his fame began.

What would have been the condition of the human race except for the doctors? They have saved so many. According to the theory of Malthus there are too many of us, not too many doctors, of course, but too many people, and this doctrine as treated in a recent article in the *North American Review* by the distinguished president of Brown University shows that at the present ratio of the increase of births over deaths, that the time will come, perhaps not more than five hundred years in the future, when there will be hardly standing room on the earth. But, thanks to the doctors, the standing room that is still left is made a good deal more comfortable and agreeable all around.

Of course, we know that everybody must die, but the desire of the human family seems to be to put off that event as long as possible. Let me give you two pictures, which are from real life. The first was in the city of Baltimore. A young woman was dying. She knew it. She had two little children. She called for them and kissed them both good night, as was her custom, and then said *that* was "good night," now this is "good-bye." Do you remember Juliet's good night to Romeo, when she stood in radiant beauty at the window, and he waited in humble submission below—do you remember her "good night, good night, parting is such sweet sorrow that I shall say good night until it be to-morrow?"

That was a pathetic scène, you think, but how different was the case of this young mother when she bade her little children good-night and then good-bye, knowing that to-morrow would find her in the everlasting beyond—beyond "the smiling and the weeping," the children orphans, the home desolate and the one inspiration and life of that home gone—to return no more.

Do you wonder that the good doctor felt emotions that he "could not express yet could not all conceal?" Do you wonder that his unbidden tears would flow? Do you wonder that he felt that he was on the very threshold between life and death and that he was in the very prescence of the Infinite? Do you wonder that he realized more fully than ever the force and fulness of Cato's soliloquy, when he saw the dagger of death and the Bible both before him:—
"This in a moment brings me to my end, but this assures me I shall never die."

But there is another picture. You have a case and it seems to be a dangerous one both for the patient and for yourself. You would like to have some professional brother to divide the responsibility with you. It is easier to carry a burden, you know, if you have some one to carry the other end; and then you might be like one of our celebrated American humorists who said that when he was a young man he took hold of the big end of the log and did all the lifting, but he learned, after a while, that the easiest way was to take hold of the little end of the log and do all the shouting—"pull together!"

It is a good thing to divide the responsibility in any professional work, but suppose you cannot get the needed help, and then you will wish yourself to be about twenty miles in the opposite direction. But you cannot go. The case is yours and you must stay, and "face the music," if there be any. Who can descrbe your feelings at that moment! You would gladly fly to your books, but alas! the books are not there. The friends and neighbors are there however, and they bewilder you with such suggestions as will make you think with Horatio's adviser, "There are more things in Heaven and earth, Horatio, than were ever dreamed of in thy philosophy." But you cannot get away. Grim fate seems to hold you there. You cannot plead that you have another engagement and will "call again." You are bound—and bound to stay.

But after awhile—after the weary watching and waiting—after some suppressed emotions and expletives not delivered, it is all over, the patient is safe, the neighbors are satisfied and you are permitted to leave, and with a long-drawn sigh of relief you bow yourself out with a hearty amen! You are glad to get out of it and to breathe the fresh air of Heaven once more.

Ah! my brethren, did you ever feel that way, when the very skies seemed to look brighter, when the stars twinkled with a gladsome welcome, when the very trees by the wayside seemed to bow their congratulations, when the horse that you were driving seemed to rejoice with you, and you and he were in love with each other, and then the watchdog's honest bark as you draw near home, and the eye that will mark your coming and look brighter when you come, and then the sweet, serene oblivion as you lay your head upon the placid pillow—at peace with all the world.

What a contrast there is between the weeping and the smiling in a physician's life! And yet, gentlemen, the highest joy must come as the reward of *duty* faithfully performed. Sometimes the reward comes in the shape of gold or silver, whether they be "at a parity" or not, and sometimes the reward does not come that way. If you will pardon a personal allusion, I once met a man who had been owing me a long time and never paid a cent, and he voluntarily came up to my buggy and said this: "Doctor, it would give me as much pleasure this morning to pay you ten dollars as if I should profess religion." A friend to whom I related this story of man's inhumanity to man said, "The best thing that fellow can do is to profess religion at once, and then pay you the ten dollars."

But sometimes, gentlemen, the reward will come to you as to a soldier in battle. Do you remember the noble surgeon Branham of the United States service who died at Brunswick, Ga., in the past summer, the first victim of the pestilence he sought to stay? Let me read you what one of our Georgia papers said of him, "*The Albany News*": "A brave death was his. He died all alone, away from those he loved and who loved him. Poor Branham! in the service of his country he died; responding to the call of his country he yielded up his life. No soldier in battle ever did more valiant service than he."

That was a beautiful tribute to a noble man. You may talk about heroism in battle, when the roar of cannon and the rattle of rifles and the companionship of comrades will urge you to the onset for victory, but what is that to the quiet heroism of the physician who stands in the very presence of the pestilence, knowing that *he* may be the first victim, as poor Branham was!

And when oblivion shall have swept away thrones and kingdoms and republics, when every vestige of human greatness and human grandeur shall have mouldered into dust, and when the last period of time shall be gone, eternity itself shall catch the glowing theme and dwell with rapture on the name of him who in the humble discharge of his duty gave his time, his talents and even his life, for the relief of the sufferings of his fellow man. And then the "hope that springs eternal in the human breast" will be realized in its full fruition.

